# Inference of mutability landscapes of tumors from single cell sequencing data

**DOI:** 10.1371/journal.pcbi.1008454

**Published:** 2020-11-30

**Authors:** Viachaslau Tsyvina, Alex Zelikovsky, Sagi Snir, Pavel Skums

**Affiliations:** 1 Department of Computer Science, Georgia State University, Atlanta, Georgia, United States of America; 2 Department of Evolutionary and Environmental Biology, University of Haifa, Haifa, Israel; National Center for Biotechnology Information (NCBI), UNITED STATES

## Abstract

One of the hallmarks of cancer is the extremely high mutability and genetic instability of tumor cells. Inherent heterogeneity of intra-tumor populations manifests itself in high variability of clone instability rates. Analogously to fitness landscapes, the instability rates of clonal populations form their mutability landscapes. Here, we present MULAN (MUtability LANdscape inference), a maximum-likelihood computational framework for inference of mutation rates of individual cancer subclones using single-cell sequencing data. It utilizes the partial information about the orders of mutation events provided by cancer mutation trees and extends it by inferring full evolutionary history and mutability landscape of a tumor. Evaluation of mutation rates on the level of subclones rather than individual genes allows to capture the effects of genomic interactions and epistasis. We estimate the accuracy of our approach and demonstrate that it can be used to study the evolution of genetic instability and infer tumor evolutionary history from experimental data. MULAN is available at https://github.com/compbel/MULAN.

## Author summary

Cancer is a dynamical evolutionary process that unfolds in populations of tumor cells. Combinations of genomic alterations of these cells affect their replication and survival. In particular, intra-tumor rates of mutation and genetic instability are often significantly higher than the normal rate. The impact of combinations of gene alterations on the genetic instability of cancer cells could be highly non-linear. In this paper, we present a computational approach called MULAN, that allows for estimation of instability rates inside heterogeneous intra-tumor populations shaped by such non-linear genetic interactions. To achieve this, we make use of single-cell sequencing, that allows to capture exact cancer clones rather than just individual mutations. We demonstrate the accuracy of our approach and show how it could be applied to experimental tumor data to study the evolution of genetic instability and infer evolutionary history. The proposed method can be used to provide new insight into the evolutionary dynamics of cancer.

This is a *PLOS Computational Biology* Methods paper.

## 1 Introduction

Cancer is a dynamical evolutionary process in the heterogeneous population of subclones [1–3], with clonal heterogeneity playing the paramount role in disease progression and therapy outcome [4–6]. Intra-tumor *genomic heterogeneity* originated from a variety of somatic events (e.g. SNVs, gains/losses of chromosomes) provides an evolutionary environment that facilitates the emergence of *phenotypic heterogeneity* that manifests itself in the extremely high diversity of phenotypic features within the tumor cell population [1, 2, 5, 7]. The genotype-phenotype mapping is often highly non-linear. It means that the effect of a combination of genes or SNVs is different from the joint effect of these genes or SNVs taken separately [8–10]. In cancer genomics, examples of such non-linear behaviour include synthetic lethality [8, 11], epistasis [12, 13] or genetic interactions [14, 15]. When phenotypic effects are associated with the reproductive success, they are often summarized within the concept of *fitness landscape* [16–19]. Within this concept, each genotype is assigned a quantitative measure of its replicative success (*fitness or height of the landscape*).

One of the hallmarks of cancer is the extremely high mutability and genetic instability of tumor cells, with intra-tumor rates of mutation, gain/loss/translocation of chromosomal regions and aneusomy (changes in numbers of chromosomes) often being several orders of magnitude higher than the normal rate [20–23]. Instability rates of subclones are just as heterogeneous as other phenotypic features. They are also subject to epistatic effects or genetic interactions [24]. As a result, it is reasonable to argue that the mutation or instability rates of a clonal population form a *mutability landscape*, whose structure is shaped by selection and genetic interactions.

Recent advances in sequencing technologies profoundly impacted cancer studies. Until recent years the most prevalent sequencing technology has been bulk sequencing, which produces admixed populations of cells. However, the most promising recent technological breakthrough was the advent of single-cell sequencing (scSeq). In the context of the current study, one of the most important advantages of scSeq is its ability to reliably and accurately distinguish exact cancer clones rather than just SNVs. It allows to study composition and evolution of intra-tumor clone populations at the finest possible resolution and take into account complex topological properties of tumor fitness and mutability landscapes, including those associated with non-linear effects.

A rich arsenal of available phylogenetic models and tools has been applied to scSeq data for solving the first important goal of reconstructing the phylogeny of cancer subclones assuming first infinite site model and then exploring more realistic but challenging models allowing recurrent or backward mutations [7, 25–28]. These advances give an opportunity to address the next important challenge: use reconstructed phylogenies to infer quantitative evolutionary parameters for cancer lineages, which can give cancer researchers a statistically and computationally sound evaluation of the effects of particular mutations or their combinations [19, 29, 30]. This problem is of paramount importance, especially for the design of efficient treatment strategies in the context of personalized medicine [8, 29, 31–34]. However, in contrast to the phylogenetic inference, very few computational tools for assessment of cancer evolutionary parameters are currently available [19, 29, 30]. In particular, several studies recently addressed the problem of inference of cancer fitness landscapes [18, 35]. In this paper, we expand the cancer evolutionary analysis toolkit by proposing a computational method for *inference of mutability landscapes and quantification of genetic instability* within clonal cancer populations.

Standard strict molecular clock-based models [36], that assume constant mutation rates, do not accurately reflect the inherent heterogeneity of cancer clone populations. Relaxation of rate constancy in the form of so-called relaxed molecular clock [37, 38] or genomic universal pacemaker [39, 40] was already introduced in other evolutionary settings such as evolution of species [38, 39] or epigenetic aging [41]. However, intrinsic heterogeneity of tumor clonal populations pose additional challenges for rate inference that should be addressed by the methods specifically tailored to cancer settings. The major challenges could be summarized as follows.

First, many currently available methods assume that closely related organisms have similar evolutionary rates [37, 42, 43] (autocorrelation property) or that rates of different genes are synchronized (genomic universal pacemaker model). In contrast, the genomic stability of individual cells is controlled by multiple molecular mechanisms for DNA damage surveillance, detection, and repair. Disruption or dysregulation of any of these mechanisms could result in different degrees of genomic instability [44]. Thus, it could be expected that mutability landscapes of intra-tumor populations are significantly more rugged than those of species or individual organisms.

**Fig 1:**
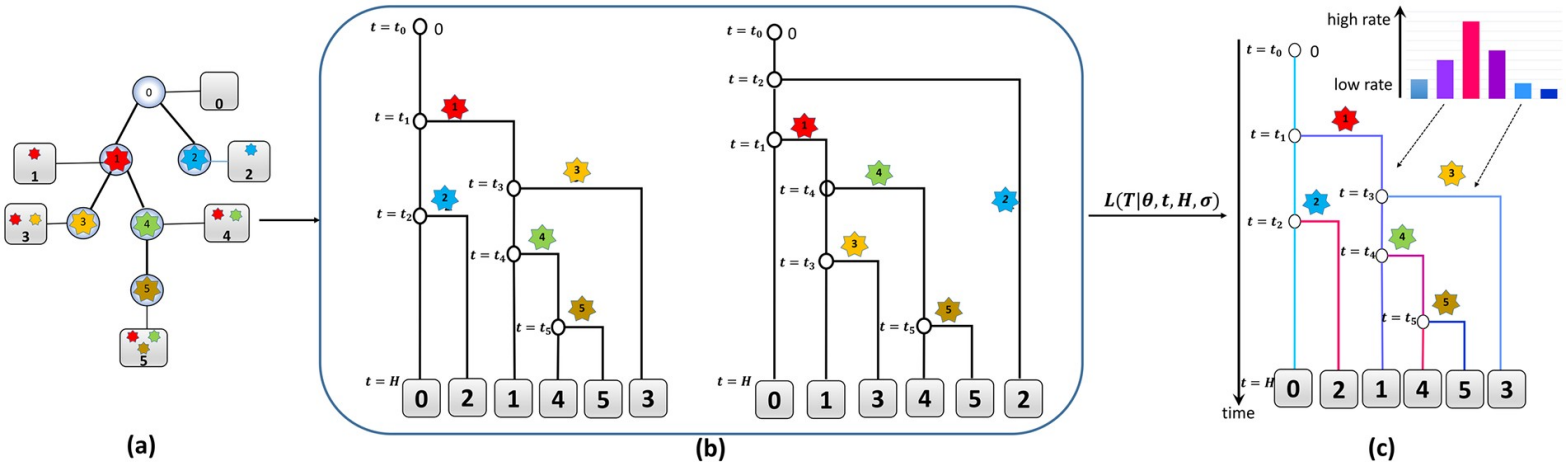
Algorithm for the maximum likelihood inference of mutability landscape. (a) Mutation tree *T*. (b) Two binary phylogenies *B*_1_(*T*) and *B*_2_(*T*) corresponding to two different orders of events *t*_0_ < *t*_1_ < *t*_3_ < *t*_2_ < *t*_4_ < *t*_5_ < *H* and *t*_0_ < *t*_2_ < *t*_1_ < *t*_4_ < *t*_3_ < *t*_5_ < *H*. Each internal vertex is labeled with its time stamp, thus resulting in the same mutation tree *T*. Each branch (*t*_*i*_, *t*_*j*_) is labeled by the leaf-subclone on the vertical line through its endpoint *t*_*j*_. All leaves have the sampling time stamp *t* = *H*. (c) Maximum Likelihood phylogeny and mutability landscape. Mutation rates along the branches corresponding to different subclones are highlighted in different colors.

Second, reconstruction of mutation rate heterogeneity via phylogenetic inference is more challenging for cancer populations than for species or organisms. Indeed, the estimation of mutation rates requires estimation of times of mutation events. The standard model for such timing is a binary phylogenetic tree, whose internal nodes represent these events and leafs correspond to sampled subclones. The timing is complicated by *polytomies* (ambiguities in order of bifurcations) that should be resolved for the inference. In cases when the expected number of mutations between a parent and its offspring is comparatively large, polytomies are relatively rare, and evolutionary distances between species provide prior information about the order of bifurcations. For the cancer subclonal populations, multiple subclones are usually at the same distance from their common parent (Fig 1), thus making polytomies extremely wide-spread. In addition, most existing approaches for single-cell cancer phylogenetics [7, 25–28, 45–50] use character-based *mutation trees* rather than binary phylogenetic trees (Fig 1). The internal nodes of a mutation tree represent mutations, leafs represent subclones, and each subclone have mutations on its path to the root. For such trees, resolution of polytomies is equivalent to finding the orders of sibling nodes, and it is crucial for the mutation rate estimation.

Finally, in established models, changes in genetic instability rates are usually associated with individual mutations. In contrast, a more accurate model would associate them with subclones, which allow capturing the effects of epistasis, including pairwise synthetic lethality, which explains cancer driver genes’ tissue specificity [8]. In general, a combined effect of several mutations cannot be explained by a linear regression model, so it is necessary to take into account the entire subclone for estimation of the mutation rate.

Here we propose MULAN (MUtability LANdscape inference)—a likelihood-based method for inference of mutability landscapes of cancer subclonal populations from single-cell sequencing data. It utilizes the partial information about the orders of mutation events provided by cancer mutation trees reconstructed from scSeq data and extends it by inferring full evolutionary history and mutability landscape of a tumor. To the best of our knowledge, it is one of the first methods specifically tailored to the cancer clone populations and scSeq data and aimed at addressing the aforementioned challenges. In particular, previously published tool SiFit [51] performs a phylogenetic inference, which includes an estimation of deletion and loss of heterogeneity rates, but these rates are assumed to be the same for all subclones. It should be noted that our method infers mutation rates of subclones rather than individual genes, thus making it possible to use the obtained results to detect and quantify genomic interactions and epistasis.

## 2 Materials and methods

### 2.1 Model

**Time-aware phylogenetic model.** scSeq data are usually represented as a 0-1 matrix in which rows correspond to sequenced cells, and columns correspond to cancer mutations. The set of ones of each row represents a *mutation profile* of a cell. Following most existing approaches for cancer phylogenetics [7, 25–28, 45–50], our basic cancer cell evolutionary model will be a *mutation tree* *T* = (*V*_*T*_, *E*_*T*_) with the vertex 0 ∈ *V*_*T*_ being the root, the internal nodes of a mutation tree representing mutations connected according to their order of appearance during the tumor evolution, the leaves correspond to the sampled subclones and the mutation profile of each cell being defined by the set of mutations on its path to the root (Fig 1A). In what follows, we assume that the *i*th subclone is attached to the internal node *i* and does not consider the leaves explicitly. The mutation tree *T* reconstructed using one of the existing methods from scSeq data constitutes and input of our algorithm. Note that *T* does not have to be a perfect phylogeny, and can contain both repeated mutations and mutation losses.

Next, we extend the phylogenetic model by accounting for times of mutation events. The mutation tree *T* provides a *partial* information about these times, as it establishes the order of mutation appearances along each path, but does not do it for sibling mutations. Therefore we need to consider a *binary phylogenetic tree* *B*(*T*) corresponding to the mutation tree *T*. The tree *B*(*T*) is defined as follows (see Fig 1):

(a) The root represents a subclone at the beginning of cancer lineage evolution.
(b) Each internal node is labeled by timestamp *t* = *t*_*i*_ representing the birth event of the offspring subclone *i*,
(c) Each leaf *i* = 0, …, *n* represents the sampling event of the subclone *i*. The tree *B*(*T*) is usually assumed to be ultrametric, i.e., all leaves are sampled simultaneously (although the model is generalizable to the non-ultrametric case, as discussed below). *H* will further denote the sampling time. Note that this value is relative, as the birth time of the root is assumed to be 0.
(d) Each edge (*t*_*i*_, *t*_*j*_) is labeled by the parent subclone of the corresponding mutation event (on Fig 1 it is the leaf *k* on the vertical through the endpoint *t*_*j*_).
(e) The orders of birth events in *B*(*T*) and mutation events in *T* agree with each other

The topology of a binary phylogeny *B*(*T*) is uniquely determined by the orderings $\sigma_{i}=\left(\sigma_{i,0},\sigma_{i,1},...\sigma_{i,d_{i}}\right)$ of the offsprings of each node *i* = 0, 1, …, *n* in the mutation tree *T*, where *d*_*i*_ is the degree of the *i*-th node in *T*. As a result, for a given mutation tree there are usually several corresponding binary phylogenies. An example of a mutation tree *T* and the corresponding binary phylogenies *B*_1_(*T*) and *B*_2_(*T*) is shown in Fig 1. The trees *B*_1_(*T*) and *B*_2_(*T*) correspond to two different plausible orders of mutation events.

**Mutability landscape likelihood model.** Next, we bring in variable mutation rates and introduce the likelihood function. We consider the **mutability landscape evolutionary model** describing subclone evolution with the underlying time-aware model similar to the model described in [52]. In this model, the appearance of mutations in each subclone is a Poisson process and time intervals between consecutive events follow the Erlang distribution. Specifically,

(a) each subclone *k* has a mutation rate *θ*_*k*_,
(b) the probability of each edge between internal nodes *e* = (*t*_*i*_, *t*_*j*_) labeled by *k* in the binary evolutionary tree is calculated as $p\left(e\right)=\theta_{k}^{2}\left(t_{j}-t_{i}\right)e^{-\theta_{k}\left(t_{j}-t_{i}\right)}$,
(c) the probability of each edge between an internal node and a leaf *e* = (*t*_*i*_, *t*_*j*_) labeled by *k* in the binary evolutionary tree is exponential and is calculated as $p(e)=\theta_{k}e^{-\theta_{k}(H-t_{i})}$.

The total probability of the tree *B*(*T*) equals *p*(*B*(*T*)|*θ*, *t*) = ∏_*e*∈*E*(*B*(*T*))_ *p*(*e*).

The described model is used to find mutability landscapes jointly with the most likely binary phylogeny *B*(*T*). We first consider the following optimization problem:

**Given:** A mutation tree *T* = (*V*_*T*_, *E*_*T*_) with mutations {0, …, *n*} ∈ *V*_*T*_ and vertex outdegrees *d*_0_, …, *d*_*n*_.

**Find:** Mutation rates $\theta ={\left(\theta_{i}\right)}_{i=1}^{n}$, times of occurrence $t={\left(t_{i}\right)}_{i=1}^{n}$ of each mutation *i* = 1, …, *n* and the sampling time *H* that maximize the probability *p*(*T*|*θ*, *t*, *H*, *σ*) of the tree *T* given the model parameters.

As noted above, setting the phylogeny *B*(*T*) is equivalent to setting the family of offspring orderings *σ* = (*σ*_1_, …, *σ*_*n*_). For a given ordering family *σ* we have

$$\begin{array}{c} p\left(T|\theta ,t,H,\sigma \right)=\prod_{i=0}^{n}\left(\prod_{j=1}^{d_{i}}\theta_{i}^{2}\left(t_{\sigma_{i,j}}-t_{\sigma_{i,j-1}}\right)e^{-\theta_{i}\left(t_{\sigma_{i,j}}-t_{\sigma_{i,j-1}}\right)}\right)\theta_{i}e^{-\theta_{i}\left(H-t_{\sigma_{i,d_{i}}}\right)} \end{array}$$ (1)

After the straightforward simplifications, the log-likelihood *L*(*T*|*θ*, *t*, *H*, *σ*) can be written as follows:

$$\begin{array}{c} L\left(T|\theta ,t,H,\sigma \right)=\sum_{i=0}^{n}\theta_{i}t_{i}+\sum_{i=0}^{n}\sum_{j=1}^{d_{i}}\log\left(t_{\sigma_{i,j}}-t_{\sigma_{i,j-1}}\right)-\left(\sum_{i=0}^{n}\theta_{i}\right)H+\sum_{i=0}^{n}\left(2d_{i}+1\right)\log\left(\theta_{i}\right), \end{array}$$ (2)

where *t*_0_ = 0, 0 ≤ *t*_*i*_ ≤ *H*, *i* = 1, …, *n*.

Our goal is to find an optimal ordering σ*, times t*, sampling time H*, and mutation rates θ* by solving the following maximum likelihood problem:

$$\begin{array}{c} \left(\theta^{*},t^{*},H^{*},\sigma^{*}\right)={\text{argmax}}_{\left(\theta ,t,h,\sigma \right)}L\left(T|\theta ,t,H,\sigma \right) \end{array}$$ (3)

Note that we usually assume that the rate *θ*_0_ is fixed (for example, to the value corresponding to the normal tissue).

The likelihood function (2) is non-linear and all nodes effectively contribute to it. This makes straightforward utilization of standard methods based on dynamic programming to solve the problem (3) is challenging. Indeed, the model implies that there exists a certain dependency between birth times of sibling subclones since they belong to the same time interval. Suppose that a subclone *i* mutated twice during the time between its birth and sampling. Although the two acquired mutations are independent and distributed uniformly at random between *t* = *t*_*i*_ and *t* = *H*, the expected birth times of two corresponding offsprings are *t*_*i*_ + (*H* − *t*_*i*_)/3 and *t*_*i*_ + 2(*H* − *t*_*i*_)/3 rather than *t*_*i*_ + (*H* − *t*_*i*_)/2. The effect of such non-linear properties of the model could be illustrated using an example on Fig 1. Intuitively, clone 1 produced two offsprings, while clone 2 produces zero offsprings. This imbalance can be explained in two ways: either (i) the clone 2 has a higher mutation rate, or (ii) clone 1 was born early and had time to accumulate mutations while clone 2 was born late and didn’t have time to accumulate mutations. When assessing these two alternatives, other clones also come into play. For example, the alternative (ii) means (a) the longer interval between the birth of clone 1 and birth of clone 2—the likelihood of such interval depends on the mutation rate of the parent clone 0; (b) the longer interval between the birth of clone 1 and the sampling—the likelihood of such interval depends on the mutation rates of the descendants of 1. Maximum likelihood inference allows us to choose between these alternatives.

In many real settings the realistic mutation rates are subject to constraints. We account for these considerations by adding to the model a prior probability *p*(*θ*). In this case, we utilize lasso regression-type approach, i.e. we solve the problem (3) under the constraint *l*(*θ*) = log(*p*(*θ*)) ≥ *l*_0_. The simplest prior assumes that the rates are distributed uniformly on the segment [*θ*_min_, *θ*_max_]. Assuming that genetic instability increase events are not frequent, we are also particularly interested in the models with the limited number of such events. In *s-model*, we assume that the rate changes in at most *s* vertices of the mutation tree. When *s* > 0, we assume that one of these rates is the normal rate and, therefore, is fixed.

Finally, we note that it is straightforward to generalize the model to the case when the tumor cells are sampled at different time points. It can be done by allowing different model-based sampling times *H*_*i*_ and setting the differences between them equal to the differences between actual sampling times.

### 2.2 Algorithms

To describe the algorithms and derive the associated mathematical claims, we will use the following notations: *T*^*k*^ is the subtree of *T* with the root *k*; *d*_*k*_ is the degree of the node *k* in *T*; *n*_*k*_ = |*V*(*T*^*k*^)|; *θ*^*k*^ is the collection of mutation rates of the vertices in *T*^*k*^ and Θ_*k*_ = ∑_*j*∈*V*(*T*^*k*^)_ *θ*_*j*_.

**A. The case without a prior *p*(*θ*).** In this case, we propose to solve the problem using an expectation-maximization approach described by Algorithm 1. This algorithm takes as an input the mutation tree *T*, feasible rates segment [*θ*_min_, *θ*_max_] and initial mutation rates *θ* = *θ*^0^, and produce as an output the mutation rates θ*, times t*, sampling time H* and orderings σ* that are supposed to maximize *L*(*T*|*θ*, *t*, *H*, *σ*). The algorithm is described as follows:

**Algorithm 1. EM algorithm for mutability landscape inference**

**Repeat** the following steps until convergence:

**M step:** for given *θ*, find *t*, *H* and *σ* maximizing *L*_*T*,*θ*_ = *L*(*T*|*θ*, *t*, *H*, *σ*) using Algorithm 2.
**E step:** for times *t* and *H*, find the expected rates:
  $$\begin{array}{c} \theta_{i}=\frac{d_{i}}{H-t_{i}} \end{array}$$ (4)

Next, we describe how M step is carried out. In what follows, we formulate several claims forming the foundation of our approach, and provide their proofs in the Subsection 2.3. For the fixed orderings *σ* and rates *θ*, (3) is a convex optimization problem with linear constraints, and thus it can be efficiently solved using standard techniques [53]. However, orderings *σ* introduce discontinuity to the objective and discretize the problem, thus making it computationally hard. The number of possible orderings *σ* is equal to $\prod_{i=0}^{n}d_{i}!$, which makes an exhaustive search over the space of all orderings infeasible. Therefore our goal is to optimize the search. Specifically, we employ the following dynamic programming approach:

**Algorithm 2. Algorithm to find optimal orderings and times, when rates *θ* are fixed**

**Input:** mutation tree *T* with the root 0 and its children 1, …, *d*, mutation rates *θ*

**Output:** times t*, sampling time H* and orderings σ* maximizing *L*_*T*, *θ*_

**1.** Recursively find optimal orderings $\sigma_{k}^{*}$ for the subtrees *T*^*k*^, *k* = 1, …, *d*.

**2.** Perform an exhaustive search over the set of permutations of (1, …, *d*). For each generated permutation *σ*_0_, we solve the problem (2) with the orderings $\sigma =\left\{\sigma_{0}\right\}\cup_{k=1}^{d}\sigma_{k}^{*}$ subject to the constraints $\frac{d_{i}}{\theta_{\max}}\leq H-t_{i}\leq \frac{d_{i}}{\theta_{\min}}$ as a convex optimization problem, and update the current best solution, if necessary. The constraints ensure that the rates calculated at each iteration of EM belong to the feasible interval.

The worst-case running time of Algorithm 2 is $O(\sum_{i=0}^{n}T\left(n_{i}\right)\cdot d_{i}!)$, where *T*(*n*_*i*_) is the running time of a numerical convex optimization algorithm with *n*_*i*_ variables. It makes the algorithm scalable for the majority of real cases when vertex degrees are not high. However, the optimality of solutions produced by Algorithm 2 is not immediately clear, and its analysis requires deeper understanding of the properties of the optimization problem (3). Such properties are established by Lemma 1 and Theorem 1. Consider the restricted version of the problem (3) with the fixed rates *θ* and the sampling time *H*:

$$\begin{array}{c} L_{T,\theta }\left(H\right)=\max_{\sigma ,t}L\left(T|\theta ,t,H,\sigma \right). \end{array}$$ (5)

Suppose that 1, …, *d* are the children of the root 0 of *T*. Then the following recurrent relation holds:

**Lemma 1.**

$$\begin{array}{c} L_{T,\theta }\left(H\right)\approx \max_{\sigma_{0}}\max_{t_{1},...,t_{d}}(H\sum_{k=1}^{d}\Theta_{k}t_{k}+\sum_{k=1}^{d}\log\left(t_{k}-t_{k-1}\right)+\sum_{k=1}^{d}n_{k}\log\left(1-t_{k}\right)+\sum_{k=1}^{d}L_{T^{k},\theta^{k}}\left(\left(1-t_{k}\right)H\right))-\\ -\Theta_{0}H+n\log\left(H\right)+\sum_{i=0}^{n}\left(2d_{i}+1\right)\log\left(\theta_{i}\right), \end{array}$$ (6)

*where the maximum is taken over permutations σ*_0_ *of* 1, …, *d* *and over* $t_{1},...,t_{d}\in R$ *such that* 0 ≤ *t*_*i*_ ≤ 1.

The relation (6) can serve as a basis for dynamic programming algorithm. However, it is not guaranteed yet that such algorithm will be efficient. Indeed, it is theoretically possible that the values of the functions *L*_*T*^*k*^,*θ*^*k*^_ are achieved on different orderings for different arguments, thus forcing the algorithm to store an exponential number of subproblem solutions. However, the following Theorem 1 guarantees that Algorithm 2 is exact, when *H* is large enough.

**Theorem 1**. For all large enough H, the optimal ordering σ* *that maximizes* (5) *is the same. It has the form* $\sigma^{*}=\left\{\sigma_{0}^{*}\right\}\cup_{k=1}^{d}\sigma_{k}^{*}$, *where* $\sigma_{k}^{*}$ *are optimal orderings of subtrees T*^*k*^ and $\sigma_{0}^{*}$ *is the permutation of* 1, …, *d that maximizes* (6).

**B. The case with a prior *p*(*θ*).** The simplest prior assumes that the rates are distributed uniformly on the segment [*θ*_min_, *θ*_max_]. For this model, initial numerical experiments suggest that the selection of the initial solution in the feasible segment ensures convergence of the EM algorithm to the feasible solution. For more complex priors, we utilize specially enhanced Markov Chain Monte Carlo (MCMC) sampling from the rates distribution that will allow for more efficient traversing of the solution space than the default approach. In particular, for *s*-model, each feasible solution could be represented by the subset *X* ⊆ *V*(*T*) of *s* internal vertices corresponding to rate change events together with the collection of *s* + 1 rates corresponding to the connected components of *T*\*X*. Then MCMC draws the new rate from the normal distribution centered on the current rate, while new subset *X*′ is drawn from the 1-flip neighborhood of the current subset *X* [54] (i.e. *X*′ = (*X* \ {*u*}) ∪ {*v*} for some *u* ∈ *X*, *v* ∈ *V*(*T*) \ *X*).

### 2.3 Mathematical foundations of the algorithms

In this subsection we prove Lemma 1 and Theorem 1. Due to the space limit, we present the general outline of the proofs and omit some particularly technical details. Let *D*[*k*] = *V*(*T*^*k*^) and *D*(*k*) = *V*(*T*^*k*^) \ {*k*} be the closed set of descendants and set of descendants of *k*, respectively.

**Proof of Lemma 1.** After variable substitution *t*_*i*_ ≔ *t*_*i*_/*H*, maximization of (2) is equivalent to the maximization of

$$\begin{array}{c} L^{\prime }\left(T|\theta ,t,H,\sigma \right)=H\sum_{i=0}^{n}\theta_{i}t_{i}+\sum_{i=0}^{n}\sum_{j=1}^{d_{i}}\log\left(t_{\sigma_{i,j}}-t_{\sigma_{i,j-1}}\right)-\Theta_{0}H+n\log\left(H\right)+\sum_{i=0}^{n}\left(2d_{i}+1\right)\log\left(\theta_{i}\right), \end{array}$$ (7)

subject to the constraints *t*_1_ = 0, 0 ≤ *t*_*i*_ ≤ 1, *i* = 2, …, *m*.

Suppose that the rates *θ*, the sampling time *H* and the family of orderings *σ* = (*σ*_0_, *σ*^1^, …, *σ*^*d*^) are fixed. Consider the partial likelihood $M\left(T|\theta ,t,H,\sigma \right)=H\sum_{i=0}^{n}\theta_{i}t_{i}+\sum_{i=0}^{n}\sum_{j=1}^{d_{i}}\log\left(t_{\sigma_{i,j}}-t_{\sigma_{i,j-1}}\right),$ which constitutes the part of the total likelihood (7) that depends on *t* and *σ*. Using simple arithmetic transformations, we get

$$\begin{array}{c} M\left(T|\theta ,t,H,\sigma \right)=H\sum_{k=1}^{d}\Theta_{k}t_{k}+\sum_{k=1}^{d}\log\left(t_{k}-t_{k-1}\right)+\sum_{k=1}^{d}n_{k}\log\left(1-t_{k}\right)+\\ +\sum_{k=1}^{d}(\left(1-t_{k}\right)H\sum_{i\in D(k)}\theta_{i}\frac{t_{i}-t_{k}}{1-t_{k}}+\sum_{i\in D[k]}\sum_{j=1}^{d_{i}}\log(\frac{t_{\sigma_{i,j}}-t_{k}}{1-t_{k}}-\frac{t_{\sigma_{i,j-1}}-t_{k}}{1-t_{k}})) \end{array}$$ (8)

Change of variables $t_{i}:=\frac{t_{i}-t_{k}}{1-t_{k}}$, *i* ∈ *D*[*k*] yields

$$\begin{array}{c} M_{T,\sigma }\left(H\right)\approx \max_{t_{1},...,t_{d}}(H\sum_{k=1}^{d}\Theta_{k}t_{k}+\sum_{k=1}^{d}\log\left(t_{k}-t_{k-1}\right)+\sum_{k=1}^{d}n_{k}\log\left(1-t_{k}\right)+\sum_{k=1}^{d}M_{T_{k},\sigma^{k}}\left(\left(1-t_{k}\right)H\right)) \end{array}$$ (9)

Thus, the relation (6) follows.

Now, let *M*_*T*,*σ*_(*H*) = max_*t*_ *M*(*T*|*θ*, *t*, *H*, *σ*) and *M*_*T*_(*H*) = max_*σ*_ *M*_*T*,*σ*_(*H*). Theorem 1 directly follows from the following lemma:

**Lemma 2**. *M*_*T*,*σ*_(*H*) ≈ *a*_*T*_ *H* − *b*_*T*_ log(*H*) + *c*_*T*,*σ*_, *where a_T_ and b_T_ are constants depending only on T, and c_T,σ_ is a constant depending on both T and σ*.

*Proof*. We will prove the lemma by induction. Suppose without loss of generality that *d* is the outdegree of the root 0 of *T*, 1, …, *d* are its children and the ordering *σ*_0_ has the form *σ*_0_ = (0, 1, …, *d*)).

a) Suppose that *T* is a star (i.e. it has 1 internal node and *d* leafs). Then we have *σ* = (*σ*_0_), $n_{k}=a_{T_{k}}=0$ and Θ_*k*_ = *θ*_*k*_ for all *k* = 1, …, *d*. For the objective we have $M\left(T|\theta ,t,H,\sigma \right)=H\sum_{k=1}^{d}\theta_{k}t_{k}+\sum_{k=1}^{d}\log\left(t_{k}-t_{k-1}\right)$, where *t*_0_ = 0. Karush-Kuhn-Tucker (KKT) optimality conditions for *t* have the following form:
  $$\begin{array}{c} \begin{array}{c} H\theta_{k}+\frac{1}{t_{k}-t_{k-1}}-\frac{1}{t_{k+1}-t_{k}}=0,\;\;\;k=1,..,d-1,\\ H\theta_{d}+\frac{1}{t_{d}-t_{d-1}}-\mu_{d}=0,\;\;t_{d}=1, \end{array} \end{array}$$ (10)
  where *μ*_*d*_ is the dual variable corresponding to the constraint *t*_*d*_ ≤ 1. After multiplying the *k*th equation by *t*_*k*_ and summing the obtained equations we get $H\sum_{k=1}^{d}\theta_{i}t_{i}=\mu_{d}-d$. Furthermore, (10) yield that $t_{k}-t_{k-1}=1/\left(\mu_{d}-H\sum_{i=k}^{d}\theta_{i}\right)$. These identities imply the following formula for *M*_*T*,*σ*_(*H*):
  $$\begin{array}{c} M_{T,\sigma }\left(H\right)=\mu_{d}-d-\sum_{k=1}^{d}\log\left(\mu_{d}-H\sum_{i=k}^{d}\theta_{i}\right), \end{array}$$ (11)
  where $\mu_{d}\geq H\sum_{i=1}^{d}\theta_{i}$ and *μ*_*d*_ satisfies the equation $\sum_{k=1}^{d}\frac{1}{\mu_{d}-H\sum_{i=k}^{d}\theta_{i}}=1.$ We will seek for the approximation of *μ*_*d*_ of the form $\mu_{d}=H\sum_{i=1}^{d}\theta_{i}+\varepsilon$, where *ε* > 0. Then from the equation for *μ*_*d*_ we have $\frac{1}{\varepsilon }+\sum_{k=2}^{d}\frac{1}{H\sum_{i=1}^{k-1}\theta_{i}+\varepsilon }=1$. For large *H*, we have $\frac{1}{\varepsilon }+o\left(1\right)=1$, thus implying that the good approximation is achieved when *ε* = 1. By substitution the expression for *μ*_*d*_ to (11) we get
  $$\begin{array}{c} \begin{array}{c} M_{T,\sigma }\left(H\right)=H\sum_{i=1}^{d}\theta_{i}+1-d-d\log\left(H\right)-\sum_{k=1}^{d}\log\left(\sum_{i=1}^{k-1}\theta_{i}+o\left(1\right)\right)\approx a_{T}H-b_{T}\log\left(H\right)+c_{T}, \end{array} \end{array}$$ (12)
  where $a_{T}=\sum_{i=1}^{d}\theta_{i}$, *b*_*T*_ = *d* and $c_{T}=-\sum_{k=1}^{d}\log\left(\sum_{i=1}^{k-1}\theta_{i}\right)-d+1$. The only term depending on the order *σ* here is the term $\sum_{k=1}^{d}\log\left(\sum_{i=1}^{k-1}\theta_{i}\right)$, which achieves the minimal value (thus maximizing *M*_*T*_(*H*)), when *θ*_1_ ≤ *θ*_2_ ≤ … ≤ *θ*_*d*_. Thus, the base case for the induction is proved.
b) Now suppose that *T* is not a star. By the induction hypothesis, for every subtree *T*_*i*_ the same ordering *σ*^*k*^ maximizes $M_{T_{k}}\left(H\right)$ for all *H*. These ordering also define the corresponding optimal binary phylogenies *B*_*k*_. We claim that it is possible to approximately estimate the optimal times *t*_1_, …, *t*_*d*_ and ordering *σ*_0_ recursively, if the solutions for the subtrees *T*_*k*_ are known. The following arguments slightly differ technically for the cases when *d* is a leaf or an internal vertex. We will demonstrate the scheme of the proof for the former case (the latter case could be handled similarly).

Consider the relation (6). After applying the induction hypothesis to *M*_*T*_*k*_,*σ*^*k*^_ we get the expression

$$\begin{array}{c} M_{T,\sigma }\left(H\right)\approx \max_{t_{1},...,t_{d}}(H\sum_{k=1}^{d}\Theta_{k}t_{k}+\sum_{k=1}^{d}\log\left(t_{k}-t_{k-1}\right)+\sum_{k=1}^{d}n_{k}\log\left(1-t_{k}\right)+\\ +\sum_{k=1}^{d}(a_{k}H\left(1-t_{k}\right)-b_{k}\log\left(H\left(1-t_{k}\right)\right)+c_{k})), \end{array}$$ (13)

where $a_{k}=a_{T_{k}}$, $b_{k}=b_{T_{k}}$ and $c_{k}=c_{T_{k},\sigma_{k}}$. Using the approximation log(1 − *t*_*k*_) ≈ −*t*_*k*_, we rewrite it as

$$\begin{array}{c} M_{T,\sigma }\left(H\right)\approx \max_{t_{1},...,t_{d}}(\sum_{k=1}^{d}\left(H\left(\Theta_{k}-a_{k}\right)+b_{k}-n_{k}\right)t_{k}+\sum_{k=1}^{d}\log\left(t_{k}-t_{k-1}\right))+\\ +H(\sum_{k=1}^{d}a_{k})-\log\left(H\right)(\sum_{k=1}^{d}b_{k})+\sum_{i=1}^{k}c_{k}, \end{array}$$ (14)

Let λ_*k*_ = *H*(Θ_*k*_ − *a*_*k*_) + *b*_*k*_ − *n*_*k*_ = *H*(Θ_*k*_ − *a*_*k*_) + *o*(*H*), *k* = 1, …, *d*. As in a), we will use KKT optimality conditions for *t*_1_, …, *t*_*d*_, which in this case have the following form:

$$\begin{array}{c} \begin{array}{c} \lambda_{k}+\frac{1}{t_{k}-t_{k-1}}-\frac{1}{t_{k+1}-t_{k}}=0,\;\;\;k=1,..,d-1,\\ \lambda_{d}+\frac{1}{t_{d}-t_{d-1}}-\mu_{d}=0,\;\;t_{d}=1 \end{array} \end{array}$$ (15)

where *μ*_*d*_ is the dual variable corresponding to the constraint *t*_*d*_ ≤ 1. Similarly to a), after multiplying the *k*th equation by *t*_*k*_ and summing the obtained equations we get $\sum_{k=1}^{d}\lambda_{i}t_{i}=\mu_{d}t_{d}-d$ and $t_{k}-t_{k-1}=1/\left(\mu_{d}-\sum_{i=k}^{d}\lambda_{i}\right)$. These identities imply that

$$\begin{array}{c} M_{T,\sigma }\left(H\right)\approx \mu_{d}-d-\sum_{k=1}^{d}\log\left(\mu_{d}-\sum_{i=k}^{d}\lambda_{i}\right)+H(\sum_{k=1}^{d}a_{k})-\log\left(H\right)(\sum_{k=1}^{d}b_{k})+\sum_{i=1}^{k}c_{k}. \end{array}$$ (16)

As above, we can use the approximation $\mu_{d}\approx \sum_{k=1}^{d}\lambda_{k}+1$. It implies that

$$\begin{array}{c} M_{T,\sigma }\left(H\right)\approx H(\sum_{k=1}^{d}\Theta_{k})-\log\left(H\right)(d+\sum_{k=1}^{d}b_{k})-\sum_{k=2}^{d}\log\left(\sum_{i=1}^{k-1}\left(\Theta_{i}-a_{i}\right)\right)+\sum_{i=1}^{k}\left(c_{k}+b_{k}-n_{k}\right)-d+1. \end{array}$$ (17)

In this formula, only the constant term depends on the order of vertices. Theorem is proved.

### 2.4 Quantification of rate estimation uncertainty

MULAN implements a maximum likelihood approach that uses the combination of discrete optimization and continuous optimization techniques to infer the solution that explains the observed data in the best possible way. In this, it follows the same paradigm as other recently published scSeq analysis tools [45, 55, 56]. However, given the uncertainty of the mutation tree estimation, it could be beneficial to provide errors or confidence intervals for the inferred rates. One possible way to do it is to combine MULAN with any tree topology sampling scheme by calculating mutation rates for the trees sampled from the particular posterior distribution given the scSec data (after burn-in). This procedure generates the posterior distribution of inferred mutation rates that can be used to calculate standard errors and/or confidence intervals. Here, we implemented this approach by combining MULAN with the tree sampling procedure utilized by SCITE [25].

## 3 Results

### 3.1 Simulated data

In this subsection, we report the results of validation of the proposed algorithm using simulated datasets. We simulated test examples with the numbers of mutations ranging from *m* = 70 to *m* = 150, which correspond to numbers of mutations for real single-cell sequencing data analyzed in previous studies [7, 25, 57]. For each test example, the simulation starts with the single clone without mutations and with the random mutation rate *θ*_0_. At subsequent iterations, existing clones *i* produce offspring at rates *θ*_*i*_; at each such event an existing clone *i* gives birth to a new clone *j* with the mutation rate *θ*_*j*_ uniformly sampled from the interval [*θ*_*min*_, *θ*_*max*_] (by default *θ*_*min*_ = 0.005, *θ*_*max*_ = 0.01) by acquiring a random mutation from the set {1, …, *m*}. The simulation ends when the desired number of clones is produced.

We validated the ability of MULAN to infer all three families of parameters of the model (3), i.e., the transmission rates, the times of mutation events, and the binary tree topology (or, equivalently, orderings of offspring of the mutation tree nodes). For the primary experiments, Algorithm 1 was executed with the initial mutation rates $\theta_{i}^{0}=\frac{1}{2}\left(\theta_{\min}+\theta_{\max}\right),i=1,...,m$. The following accuracy measures were used:

- Rate and time inferences were quantified by the mean absolute percentage accuracy *MAPA* = 1 − *MAPE*, where *MAPE* is the mean absolute percentage error.
- Ordering inference was quantified by the mean Kendall tau distance between true and inferred offspring orders for the nodes with outdegrees *d*_*i*_ ≥ 2.

The mutation rates of leafs were not considered, since they do not have offsprings required for reliable rate estimation.

**Fig 2:**
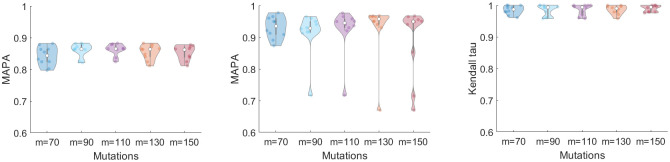
Performance of MULAN on simulated data with *n* = 70, …, 150 mutations. Left: accuracy of rate estimation. Center: accuracy of times estimation. Right: accuracy of orderings estimation.

**Fig 3:**
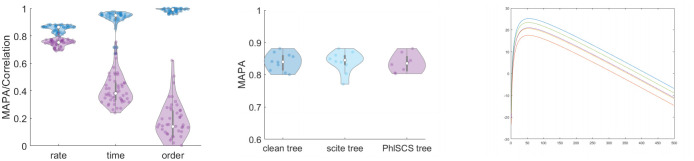
Left: accuracies of rate, time and order estimation for MULAN (blue) and MCMC algorithm (red). Center: accuracy of rate estimation (*n* = 70) for the clean data and the trees inferred by SCITE and PhISCS-BnB from noisy data. Right: likelihoods *L*_*T*,*σ*_(*H*) for different orderings *σ*. The graph demonstrates the hierarchy of orderings based on the corresponding likelihoods that remain the same for all sampling times *H*.

The results of MULAN evaluation on simulated trees are shown in Fig 2. The mean accuracies of rate, time and order inference were 0.86 (*std* = 0.02), 0.92 (*std* = 0.11) and 0.98 (*std* = 0.01), respectively. The ability of MULAN to accurately reconstruct tree topologies is particularly important, as it validates the application of MULAN to the analysis of evolutionary histories described in Subsection 3.2. The number of mutations does not have a great impact on the algorithm accuracy, possibly because the algorithm is likely to produce the optimal solution with respect to the objective (2) owing to the optimized search over the space of possible mutation orderings and the accuracy of the estimations suggested by Theorem 1. Indeed, the crucial assumption of our approach is based on Theorem 1, which establishes the hierarchy of mutation orderings that is valid for all sampling times. Although Theorem 1 operates with approximations, the experimental validation suggests that this hierarchy is always valid (Fig 3, right). Changing initial conditions to the random values uniformly sampled from the interval [*θ*_min_, *θ*_max_] does not significantly affect the results, with the mean rate, time and order inference accuracy changing to 0.83, 0.92 and 0.96, respectively.

In another evaluation experiment, we compared MULAN with an MCMC-based method, which samples from the space of tree edge lengths using the method proposed in [51], calculates birth times and orderings from these lengths and estimates mutation rates using (4). The mean accuracies of rate, time and order inference of this method were 0.72 (*std* = 0.03), 0.40 (*std* = 0.11) and 0.18 (*std* = 0.16), respectively (Fig 3, left). We also verified MULAN’s robustness to the sequencing noise and to the choice of the tumor phylogeny inference method. In that case, random errors were introduced to clone mutation profiles with *n* = 70 mutations and with 3 copies of each clone at false-negative rates *α* = 0.1 and the false positive rate *β* = 10^−5^, the mutation trees were reconstructed from these profiles using the state-of-the-art tool SCITE [25] and the recently released tool PhISCS-BnB [45, 58]. The accuracy of rate inference was affected insignificantly (Fig 3) indicating the robustness of MULAN results to the sequencing noise provided the properly selected phylogeny inference algorithm.

**Fig 4:**
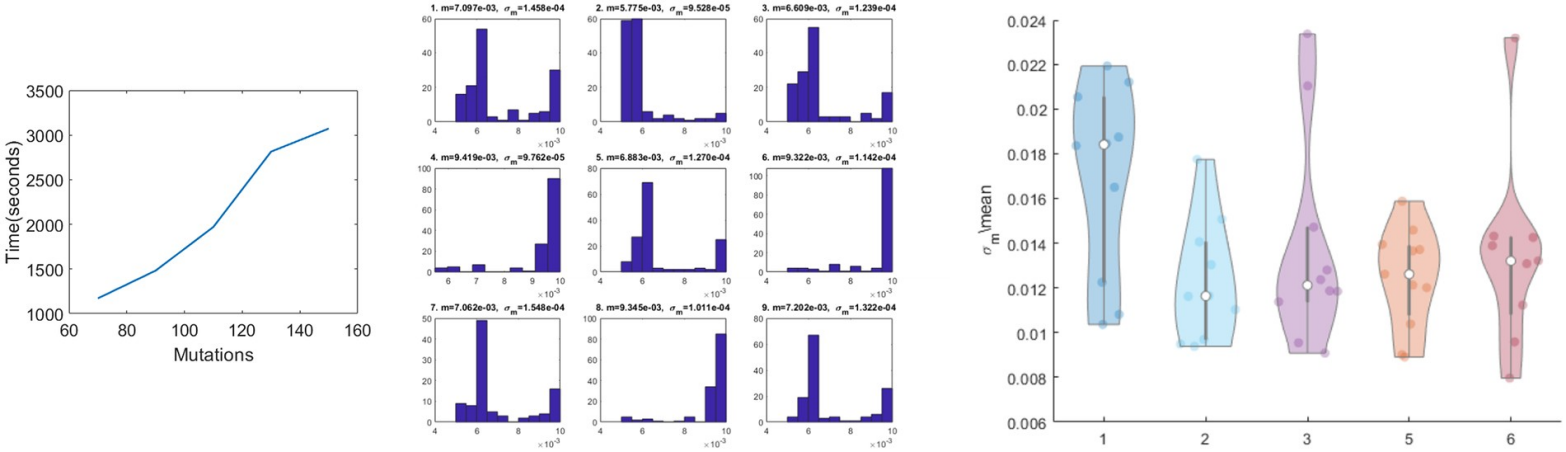
Left: algorithms’ running time. Center: the posterior distributions of inferred mutation rates for 9 selected subclones in one of the test datasets. Each small plot shows the rate distribution for the particular subclone together with the mean value *m* and the standard error *σ*_*m*_. Right: distributions of relative standard errors of rate distributions for five test datasets.

The algorithm scales polynomially with the problem size and produces the results within minutes (Fig 4, left). In the overwhelming majority of cases, EM converges within 10 iterations.

Finally, Fig 4, center and right, demonstrates the posterior distributions and relative standard errors (i.e. the standard error divided by the mean) of inferred mutation rates for several test datasets, as estimated using the method described in Subsection 2.4.

### 3.2 Experimental data

In this subsection, we used MULAN to analyze scSeq data from *JAK*2-negative myeloproliferative neoplasm [59] and from lymphoblastic leukemia [60]. The datasets contain 18, 20, 16, 10 mutations and 58, 111, 115 and 146 cells, respectively, and were analyzed as is without any modifications.

**Fig 5:**
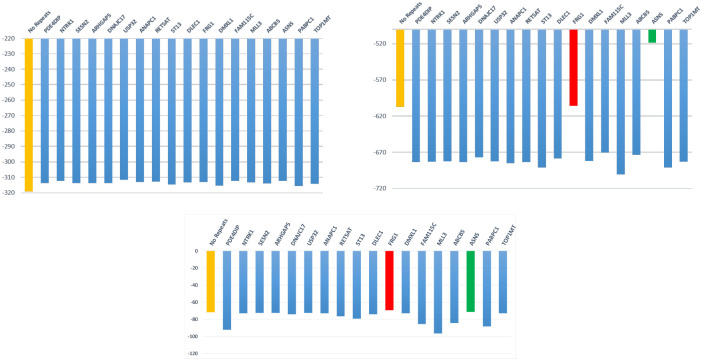
Log-likelihoods of trees with and without recurrent mutations for *JAK*2-negative myeloproliferative neoplasm. Upper left: log-likelihoods produced by infSCITE. Upper right: log-likelihoods produced by SCIFIL. Lower middle: log-likelihoods produced by MULAN.

**Analysis of evolutionary histories.** Here we used the MULAN model to assess the likelihoods of alternative tumor evolutionary histories. The datasets under consideration were used in [61] to demonstrate the violation of the infinite site assumption. For a dataset with *m* mutations, the authors of [61] used the tool infSCITE to infer the perfect phylogeny and *m* mutation trees *T*_*i*_ with one of *m* mutations *i* having a recurrence (*recurrence trees*). According to the error-based likelihood model used in [61], the recurrent trees have much higher likelihoods than the perfect phylogeny (Fig 5), thus strongly pointing to the presence of recurrent mutations. However, differences between the likelihoods of recurrence trees are of much smaller magnitude than their difference with the perfect phylogeny. It suggests that without the infinite site assumption, the number of possible alternative evolutionary histories accurately explaining the observed ScSeq data increases, and it becomes challenging to choose between by taking into account only sequencing errors. In what follows we demonstrate that evolutionary-based likelihood estimated using MULAN allows to significantly reduce the set of plausible evolutionary histories.

For each tree constructed by infSCITE, we estimated the following:

(a) the evolutionary likelihood of the most probable fitness landscape, as calculated by our recently published tool SCIFIL [18]. Roughly speaking, this likelihood measures the probability to observe given subclone frequencies when the clonal population evolutionary trajectory over the most likely inferred fitness landscape is described by the tree *T*.
(b) the likelihoods of mutation instability landscapes with three mutation rates, one of which correspond to the normal rate.

It turned out that for the analyzed dataset, mutability likelihoods and evolutionary likelihood provided an additional strong signal that allows to resolve the ambiguities present in the error-based model. It is especially visible for the *JAK*2-negative myeloproliferative neoplasm (Fig 5). There, both likelihoods point to the same two mutations *FRG*1 and *ASNS* as most probable recurrent mutations and trees *T*_*FRG*_ and *T*_*ASNS*_ as most probable trees. Only these two trees had higher likelihoods than the perfect phylogeny (even despite the fact that they define more transmission events), and their mean mutability log-likelihoods were higher than for other recurrence trees: −70.46 (*std* = 1.53) vs −78.75 (*std* = 7.79).

Independent acquisitions of mutations with confirmed cancer effects in parallel lineages potentially indicate the convergent evolution and may be suggestive of their evolutionary advantage. In this context, it should be noted that both *FRG*1 and *ASNS* have been identified in [59] as belonging to the shorter list of selected mutations having the highest likelihood of being involved in essential thrombocythemia initiation and/or progression. Furthermore, 5 out of 7 most likely repeated mutations identified by MULAN belong to that list.

For the lymphoblastic leukemia datasets, the signal was not so strong, possibly because introductions of repeated mutations did not significantly alter the topologies of the recurrence trees (see [61]), thus resulting in many of them having close mutability likelihoods. Nevertheless, even then, the correlations between evolutionary and mutability likelihoods of the trees of the 5 analyzed datasets were 0.85, 0.31, 0.96, 0.91, and 0.69, respectively, with both models agreeing on the most probable recurrence trees. The fact that the same signal was produced by two independent models can be considered as an indicator of their validity. It also suggests that the reliable inference of tumor phylogenies under the finite site assumption requires the utilization of advanced likelihood models that take into account the dynamics of cancer evolution in addition to the simpler models regulating the number and type of mutation events.

**Analysis of mutability models.** In this set of experiments, our purpose was to test the assumption that mutation rates change over the course of tumor evolution. For this purpose, we compared the single-rate model with the simplest model non-flat mutability landscape model that assumes two mutation rates. Following [61] and [39], the moldels were compared using Bayes factor *BF* [62], Akaike Information Criterion difference Δ*AIC* [63] and Bayesian Information Criterion difference Δ*BIC* [64]. In our case, these parameters are estimated as

$$\begin{array}{c} BF=exp\left(L_{2}-L_{1}\right),\;\;\;\Delta AIC=2\left(k_{1}-k_{2}\right)+2\left(L_{2}-L_{1}\right),\;\;\;\Delta BIC=\left(k_{1}-k_{2}\right)\log\left(n\right)+2\left(L_{2}-L_{1}\right), \end{array}$$ (18)

where *n* is the number of vertices of the tree *T*, *L*_1_ and *L*_2_ are maximum log-likelihoods of one-mutation and two-mutation models, and *k*_1_ = 1 and *k*_2_ = 3 are the numbers of parameters estimated by these models (the mutation rate in the former case and the two mutation rates and one rate change event in the latter case). Larger positive values of parameters indicate the preference of the two-rate model over the one-rate model. The models were compared for the perfect phylogeny *T*_*PF*_ and the two most probable recurrence trees *T*_*FRG*_ and *T*_*ASNS*_ for the *JAK*2-negative myeloproliferative neoplasm [59], as well as for the trees produced by SCITE [25] for lymphoblastic leukemia datasets [60]. For 3 out of 6 trees, the evidence for the variable mutation rate is considered as very strong (according to [62]), for 2 trees—as strong, and for one tree (*T*_*FRG*_) the evidence for any of the models was not conclusive (Table 1).

**Table 1:** Comparison of one-rate and two-rate models for experimental data.

Tree	*T*_*PF*_ [59]	*T*_*FRG*_ [59]	*T*_*ASNS*_ [59]	*T*_1_ [60]	*T*_2_ [60]	*T*_3_ [60]
*BF*	5.010 ⋅ 10^5^	1.448 ⋅ 10^1^	2.587 ⋅ 10^5^	5.037 ⋅ 10^3^	3.882 ⋅ 10^2^	9.199 ⋅ 10^1^
Δ*AIC*	26.249	5.3456	24.925	13.049	7.923	5.043
Δ*BIC*	20.358	−0.543	19.036	11.058	6.378	4.438

**Fig 6:**
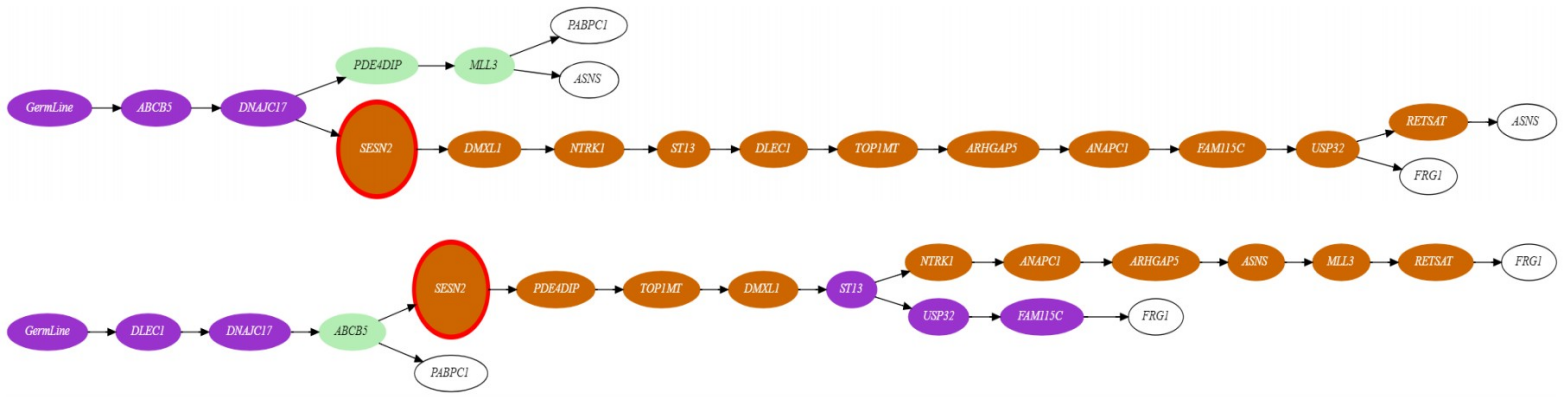
Two alternative mutation trees with the repeated mutations in *ASNS* gene (top) and *FRG*1 gene (bottom), respectively. The different mutation rates are color-coded from green (low rate) to orange (high rate). The node corresponding to the mutation in *SESN*2 gene is highlighted. Leafs (not taken into account) are highlighted in white.

**Mutability landscape of *JAK*2-negative myeloproliferative neoplasm.** For two most likely recurrent trees *T*_*FRG*_ and *T*_*ASNS*_ identified above, more detailed analysis of their mutability landscapes using the general MULAN model demonstrated that in both cases the increase in the inferred mutation rates is likely associated with the emergence of mutation in the gene *SESN*2 (Fig 6). *SESN*2 is an antioxidant activated by p53, and it is indeed known that mutations in this gene may lead to genetic instability [59]. The structures of inferred mutability landscapes for these two trees also suggests that under the maximum parsimony criterion the first tree could be considered as more plausible than the second tree, where clones revert from higher to lower rates in one of its branches.

## 4 Discussion

Genomic instability is a typical characteristic of cancer cells, which may significantly contribute to tumor progression. Another paramount feature of cancer is an extremely high intra-tumor heterogeneity, with the genomic instability being one of the traits that may significantly differ between subclones. Thus, quantification of differential mutability and genomic instability for tumors may provide valuable information for understanding mechanisms of cancer progression and the design of personalized treatment strategies. The phenomenon of heterogeneous genomic instability could be geometrically represented by a concept of *mutability landscape*, which is the analog of the classical concept of the fitness landscape. Single-cell sequencing provides an unprecedented insight into intra-tumor heterogeneity and allows us to assess and study mutability landscapes of tumors on the finest possible level of individual subclones. In this paper, we presented likelihood-based methods for the inference of mutability landscapes of cancer subclonal populations from single-cell sequencing data. Most available methods for inference of differential mutation rates are tailored to the populations consisting of relatively distant genomes. In contrast, our method is specifically tailored to the specifics of cancer clone populations that consist of highly similar but distinct genomes and takes full advantage of the information about the structure and evolutionary history of the clonal population provided by single-cell sequencing. It infers mutation rates of subclones rather than individual genes, thus making it possible to use the obtained results to detect and quantify genomic interactions and epistasis. Instead, then considering all possible cancer phylogenies, MULAN uses as a starting point, a character-based mutation tree produced by other tools. This tree represents partial information about the order of the appearance of the clones. MULAN enriches this information by reconstructing orders of the appearance of sibling clones in the tree and uses it to infer mutation rates and clone appearance times. Thus, our methods can be used jointly with available tools for cancer tree inference from scSec data, such as SCITE [25], SiFit [51], SPhyR [27] and SCARLET [56], as well as from a combination of bulk and scSec data such as B-SCITE [46] and PhISCS [45]. The latter approach could be especially useful in the context of mutation clusters resolution. Indeed, MULAN assumes by default that every mutation results in a new subclone. However, scSec-based methods sometimes infer branches of mutations whose linear ordering cannot be resolved and group them into mutation clusters. Bulk data provides information about variant allele frequencies that allows inferring the temporal order of such mutations [46]. If such data is unavailable, ambiguities in clusters could be resolved arbitrarily, but the set of inferred mutation rates of clustered nodes should be interpreted as representing the whole subpopulation rather than individual subclones.

Our experiments demonstrated that the proposed approach allows for accurate inference of mutability landscapes and can be used for the analysis of the evolutionary history for real tumors. In particular, MULAN was able to detect a mutability increase event during the evolution of *JAK*2-Negative Myeloproliferative Neoplasm, that could be linked to the mutation in the gene with known associations with genetic instability. In addition, for several analyzed tumors the evolutionary signal produced by our mutability landscape model agreed with the signal produced by an independent fitness landscape model. This fact could be considered as an indication of the validity of both models.

There are several directions for the possible expansion of the proposed computational framework. Since mutation rates are the most important parameters for the inference, it could be beneficial to marginalize the likelihood over the remaining parameters. It may require the derivation of analytical expressions and/or accurate approximations for the marginalized likelihood that allows reducing its maximization to convex programming. Another direction is the development of the joint model for the inference of mutation and replication rates of cancer subclones. In this paper, we follow the common assumption of the standard molecular clock-based methods that do not consider population sizes. This assumption is usually justified, for example, using the neutral theory of molecular evolution [65, 66], which is also applicable to cancer [67, 68]. To take into account a wider range of evolutionary scenarios, a comprehensive framework incorporating replication rate and mutation rate diversity should be developed. One of advantages of such approach is its ability to utilize the observed frequencies of sequenced clones for the inference (for example, of mutation orders). Such utilization is not straightforward [18, 69]: high frequency of a particular clone can be indicative of its earlier birth time or of its higher replication rate. To distinguish between these alternatives, an incorporation of a separate maximum likelihood framework is necessary. It potentially could be achieved, for example, by integrating MULAN with our previously published framework SCIFIL for the inference of cancer fitness landscapes [18]. Finally, MULAN was developed with targeted single-cell sequencing experiments in mind and it scales well for datasets typical for such settings. It is still scalable for whole-genome sequencing, if the mutation tree has not too many branching events. However, for more branching trees with thousands of vertices the scalability could become an issue. In that case, faster strategy for search in the space of mutation orderings should be considered.
